# Avulsion of the femoral attachment of the medial collateral ligament in the setting of knee multiligament injury: A case report

**DOI:** 10.1097/MD.0000000000018376

**Published:** 2019-12-16

**Authors:** Deming Guo, Haichi Yu, Bingzhe Huang, Xue Gao, Yanguo Qin, Xiaoning Liu

**Affiliations:** Orthopaedic Medical Center, The Second Hospital of Jilin University, Changchun, China.

**Keywords:** knee injuries, medial collateral ligament avulsion, multiligament injury, surgical repair

## Abstract

**Rationale:**

Medial collateral ligament (MCL) injury is a common sports injury. The damage mainly occurs in ligament fibers, but MCL avulsion fracture is extremely rare and only a few reports have been published.

**Patient concerns:**

Herein, we present a healthy 21-year-old man with an avulsion fracture of the MCL of the right knee sustained during snowboarding.

**Diagnosis:**

Clinical and radiographic findings confirmed the presence of an avulsion fracture at the proximal attachment of the MCL, combined with complete anterior cruciate ligament (ACL) and posterior cruciate ligament (PCL) rupture.

**Interventions:**

The patient underwent single-stage ACL, PCL reconstruction, and MCL repair.

**Outcomes:**

Two weeks after the surgery, the patient developed heterotopic ossification (HO) at the medial side of the knee, HO tended to be stable and mature at the 3-month follow-up examination. One year after the operation, the patient's knee was fully functional, stable, and pain free.

**Lessons:**

Femoral attachment avulsion fracture of the MCL is in contrast to common isolated MCL injuries. Early surgical repair is advocated for the greatest benefit. Orthopedic surgeons should keep the potential complication HO in mind and develop rational strategies for HO prevention and treatment.

## 1 Introduction

The medial collateral ligament (MCL) is the main stabilizing structure of the medial side of the knee. It resists valgus stress, provides static and dynamic stability, and assists in resisting rotational stress and anterior-posterior translation.^[1–3]^ In general, damage mainly occurs when forced valgus movements take place on a partly flexed knee with or without rotation. This is often accompanied by complete tearing of the cruciate ligament or a merge with a tear of the meniscus.^[1,4]^ Most MCL tears occur in the mid-body. An avulsion fracture that occurred at the femoral attachment was first reported in the literature in 2007.^[5]^ As far as we know, there are only 3 cases reported in the literature involving the MCL femoral attachment.^[5–7]^ Unlike previous reported cases of isolated avulsion fractures, the case presented here is of a skeletally mature athletic man who suffered from combined MCL femoral attachment avulsion fracture in addition to a complete anterior cruciate ligament (ACL) and posterior cruciate ligament (PCL) tear in a skiing accident. The patient was treated surgically and followed up for 1 year. This report is intended to provide orthopedic surgeons with a better understanding of injury types, management, surgical outcome, and specific treatment concerns. The patient provided informed written consent that the medical records and imaging data would be submitted for publication, and the Jilin university second hospital institutional review board approved the study.

## 2 Case report

A 21-year-old man was referred to the trauma center with severe knee pain from a snowboarding accident 5 days earlier after he slipped while jumping off the snowboard in the semi-flexion position of the right knee. He had immediate, severe pain and swelling of the knee, which was still unable to bear weight, with an initial treatment of long leg splint. Physical examination showed no impairments in skin integrity, moderate joint effusion, and widespread subcutaneous hematoma around the knee. The patient had significant tenderness along the course of the MCL, but there was no neurovascular deficit. The patient showed positive tibia lag sign, and grade 3 Lachman and anterior drawer test results with a soft endpoint, and grade 3 valgus stress test results. These findings collectively suggested tearing of the ACL and PCL.

**Figure 1:**
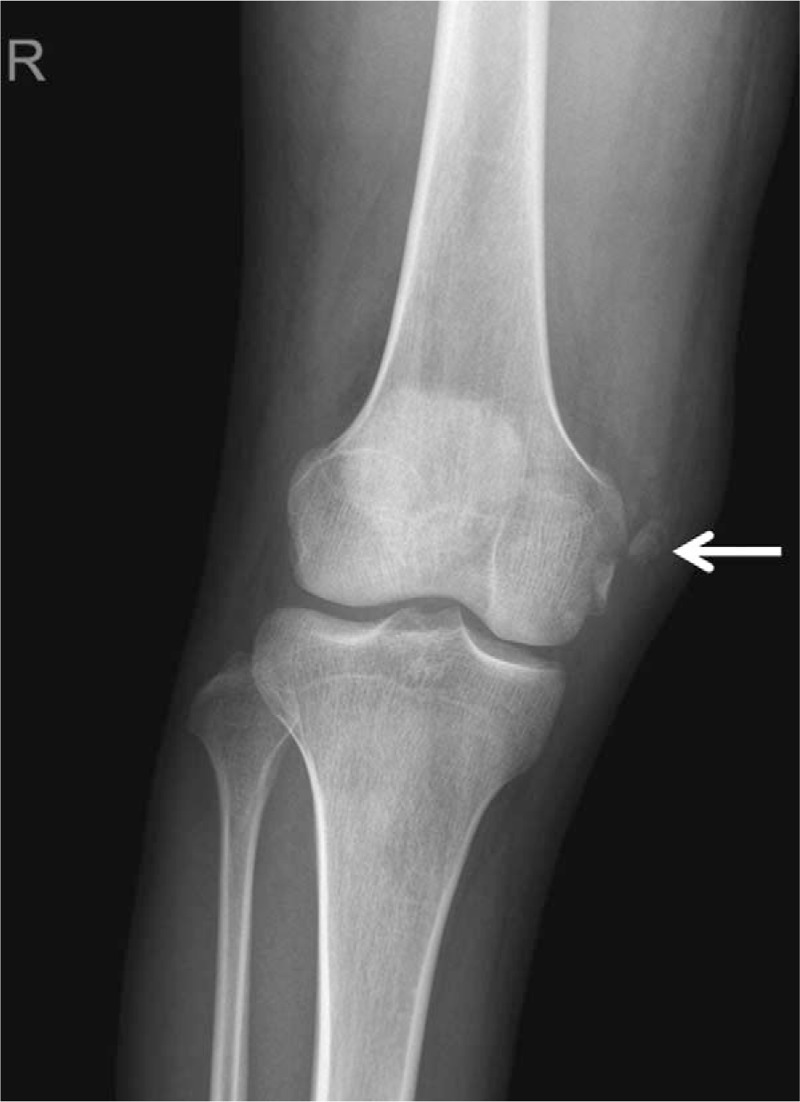
Knee anteroposterior (AP) view. Arrow indicates medial collateral ligament (MCL) femoral attachment fragment.

**Figure 2:**
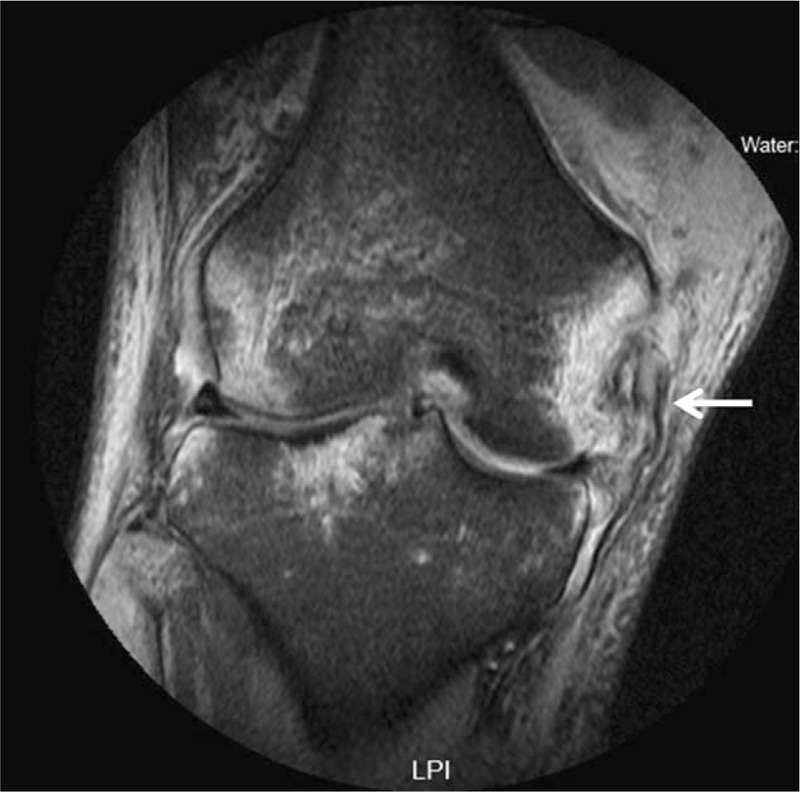
Coronal T2 fat decompression magnetic resonance imaging (MRI). Arrow indicates bony avulsion fracture with intact ligament fibers.

Plain radiographs of the knee anteroposterior and lateral views showed osseous avulsion adjacent to medial femoral condyle (Fig. 1). Magnetic resonance imaging (MRI) revealed a bony fragment at the proximal attachment of the MCL, combined with a mid-substance ACL and PCL tear (Fig. 2). These results collectively indicated MCL femoral attachment avulsion fracture in addition to a complete ACL and PCL tear.

## 3 Surgical technique

### 3.1 Patient setup and diagnostic arthroscopy

The patient signed the informed consent form, and the operation was performed under continuous epidural anesthesia. The patient was placed supine on the table with a leg holder to keep the knee flexion at 90° and so facilitate concomitant ligament reconstruction. First, diagnostic arthroscopy was performed to address the ACL, PCL lesion using standard high anterolateral and anteromedial portals.

### 3.2 Preparation of grafts

The semitendinosus and gracilis tendons were obtained from the bilateral knee with a closed-ended tendon stripper through a 2-cm oblique incision along the distal pes anserinus insertion. The PCL graft was prepared with a 4-strand semitendinosus tendon, 9.5 mm in diameter, and the ACL graft was prepared using a bilateral 4-strand gracilis tendon 8 mm in diameter. All grafts were wrapped in saline gauze after pretensioning for later use.

### 3.3 Establishment of PCL tunnel

A posteromedial accessory portal was established using an outside-in technique with an 18G spinal needle. The scope was driven through the anterolateral portal for PCL tibial insertion site visualization and stump debridement. With the knee in 90° flexion, a PCL tibia guide was introduced from the high anterior medial portal to the posterior side of the knee, and the tibial tunnel exit was located 1.5 cm below the posterior tibia plateau to establish a full-length tibia tunnel. The femoral socket was positioned relative to the anatomic center of the PCL stump. The established socket allowed the graft to enter at least 25 mm.

### 3.4 ACL tunnel placement

During the ACL reconstruction, after the tibial footprint of the ACL was located with a thermal device at 90° of knee flexion, the tibial tunnel was created using a 55° ACL tibial aimer with the tip pointed to the center of ACL tibia footprint, and the femoral socket was created using a modified transtibia technique at the center of native ACL insertion with a tunnel length of 25 mm.

### 3.5 Graft passage and fixation

After the tunnels were prepared, the PCL graft was passed through first and fixed to the media femoral condyle using an EndoButton (Smith & Nephew, Andover, MA). The same device was used to fix the femoral side of the ACL after graft passage. After pretensioning of the 2 grafts, the knee's motion was evaluated to avoid impingement and 2 interference screws were used to secure the graft at the tibial tunnel separately. Secondary fixation was achieved using a U-shape stable.

### 3.6 MCL femoral attachment avulsion fracture refixation

**Figure 3:**
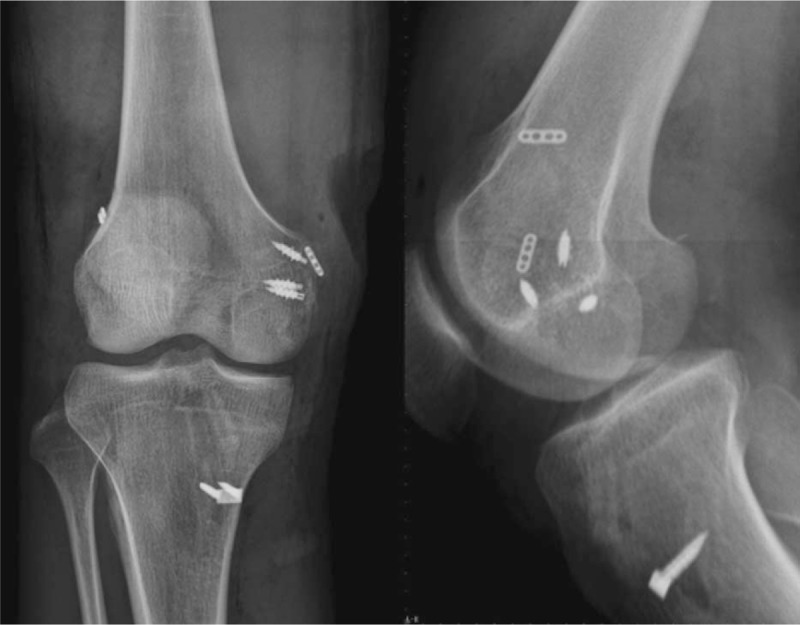
Anteroposterior (AP) and lateral radiographs immediately after surgery.

An approximately 5-cm incision centered on the medial femoral condyle was made to expose the femoral attachment of MCL with a careful dissection to the fascia layer. A large bone fragment was identified attached to the MCL, of which the MCL is intact. The repair was completed by deploying three 5.5-mm double-loaded metal suture anchors (Smith & Nephew, Andover, MA) along the bone bed in a double-row configuration (Fig. 3).

### 3.7 Postoperative rehabilitation

During the first 6 weeks after surgery, the patient wore a hinged knee brace with a slight varus mold and was allowed to perform toe touches and put some weight on the limb while walking with the brace locked in the extended position. Under other circumstances, the brace was kept loose during knee exercises. Six weeks after the operation, progressive load bearing was allowed with the brace unlocked. Strength training was initiated 12 weeks after the surgery. One year after the operation, the patient's knee was fully functional, stable, and pain free.

## 4 Discussion

Damage to the medial structure of knee is one of the most common sports injuries, accounting for more than one-third of all sports-related knee injuries.^[1,3]^ Contact sports, such as football, American football, and skiing, involve a high risk of injury to the medial side structure.^[8,9]^ Up to 80% of grade III MCL injuries are associated with knee cruciate ligament injury; a large proportion of them are ACL injuries.^[4,8]^ High-energy trauma due to motor vehicle and industrial accidents often results in multiple ligament injuries to the knee. This type of injury should be treated as dislocation of the knee, and single stage repair and reconstruction of the medial structure are always needed, associated with ACL and PCL reconstruction. In order to prevent postoperative knee stiffness, some clinicians advocate a staged approach to deal with such injuries, beginning with PCL reconstruction and medial structural repair/reconstruction followed by ACL reconstruction after 6 weeks.^[3,4,9]^

MCL avulsion fracture is uncommon in adults. Most MCL tears are fiber ruptures adjunct to the femoral origin, involving injuries to multiple ligaments of the knee. The combination of tears to the MCL, ACL, and PCL is not rare and is well recognized; almost all of these cases involve disruption of the fibers of the MCL. This possible mechanism of injury in the present case may be attributed to forceful valgus motion with external rotation of the tibia and hyperextension of the knee at the moment of the patient's fall while snowboarding.

Naik et al^[5]^ in 2007 reported an MCL avulsion fracture from both femoral and tibia attachments after a motorcycle collision in a 28-year-old man. As in our case, both the ACL and PCL were torn. The patient underwent a staged surgery with the MCL repair first, after which knee flexion >90°was achieved and staged PCL and ACL reconstruction was carried out. One year later, the patient's knee was stable with full range of motion.

In 2017 Haddad et al^[7]^ presented a case of MCL femoral avulsion fracture combined with complete rupture of the PCL in a 38-year-old man who fell during a recreational sport. They report that the patient had a painful, swollen knee, and positive valgus laxity at both 0° and 30°associated with a grade III posterior drawer test on physical examination. Radiographs revealed a bony fragment at the media femoral condyle, and MRI findings confirmed that the PCL tear was complete. The patient accepted single-stage surgery consisting of double-bundle PCL reconstruction with Achilles allograft and primary MCL repair performed with anatomical reduction of the fragment with bone anchors and washer. Passive range of motion was initiated immediately after surgery. Two years into follow-up, the patient demonstrated excellent function, a pain-free knee, and was able to return to sports activity.

**Figure 4:**
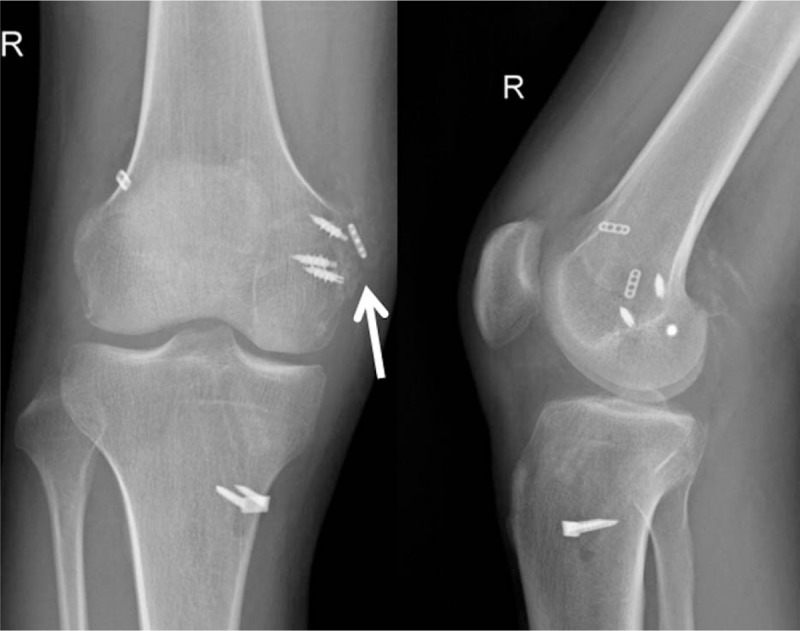
Anteroposterior (AP) and lateral radiographs at 3-month follow-up. Arrow indicates mature heterotopic ossification at the medial side of the knee.

No previous report named any complications associated with MCL femoral avulsion fracture. However, in the current case, at postoperative 2 weeks, heterotopic ossification (HO) occurred on the medial side and proceeded gradually. To slow the progression of HO, the patient was prescribed indomethacin for 6 weeks. Three months after surgery, ossification appeared mature, with a clear trabecular structure and rounded edges, and progression appeared to have ceased (Fig. 4). The cause of HO remains unclear as of this publication, and the treatment is quite challenging. Orthopedic surgeons should keep this potential complication in mind and develop rational strategies for HO prevention and treatment.

In conclusion, a thorough understanding of anatomical and biomechanical foundations of the media side knee, in conjunction with physical examination, is paramount in ascertaining the full extent of injury and to make treatment decisions. Femoral attachment avulsion fracture of the MCL combined with tearing of the ACL and PCL is extremely uncommon and very few previous cases have been described. This is in contrast to common isolated MCL injuries. Early surgical repair is advocated for the greatest benefit.

## Author contributions

**Methodology:** Bingzhe Huang, Xue Gao, Yanguo Qin.

**Conceptualization:** Deming Guo, Haichi Yu, Xiaoning Liu.

**Data curation:** Deming Guo, Haichi Yu, Xiaoning Liu.

**Investigation:** Deming Guo, Haichi Yu, Xiaoning Liu.

**Methodology:** Bingzhe Huang, Xue Gao.

**Resources:** Deming Guo, Haichi Yu.

**Writing – original draft:** Deming Guo, Xiaoning Liu.

**Writing – review and editing:** Bingzhe Huang, Xue Gao, Xiaoning Liu.
